# Meta-Analysis of ABCG2 and ABCB1 Polymorphisms With Sunitinib-Induced Toxicity and Efficacy in Renal Cell Carcinoma

**DOI:** 10.3389/fphar.2021.641075

**Published:** 2021-03-08

**Authors:** Fengjun Sun, Zhuo Chen, Pu Yao, Bangbi Weng, Zhirui Liu, Lin Cheng

**Affiliations:** ^1^ Department of Pharmacy, The First Affiliated Hospital of Third Military Medical University (Army Medical University), Chongqing, China; ^2^ Department of Pharmacy, Chongqing Emergency Medical Center, Chongqing, China

**Keywords:** renal cell carcinoma, sunitinib, ABCG2, ABCB1, polymorphism, meta-analysis

## Abstract

**Background:** ABCG2 and ABCB1 are genes related to the pharmacokinetics of sunitinib and have been associated with its toxicity and efficacy. However, the results have been controversial. This study aimed to evaluate the associations of ABCG2 and ABCB1 polymorphisms with sunitinib-induced toxicity and efficacy in renal cell carcinoma (RCC) by meta-analysis.

**Methods:** *PubMed, EMBASE, Cochrane Library*, and *Web of Scienc*e were systematically searched for studies investigating the associations of the ABCG2 rs2231142 polymorphism with sunitinib-induced toxicity and the associations of the ABCB1 rs1128503 and ABCB1 rs2032582 polymorphisms with sunitinib-induced toxicity and clinical outcomes. The associations were evaluated by effect size (ES) with 95% confidence intervals (CIs).

**Results:** Eight and five studies were included in the toxicity and efficacy analysis, respectively, including a total of 1081 RCC patients. The ABCG2 rs2231142 A allele was associated with an increased risk of sunitinib-induced thrombocytopenia and hand-foot syndrome (HFS) in Asians (ES = 1.65, 95% CI = 1.15–2.36, *p* = 0.006; ES = 1.52, 95% CI = 1.02–2.27, *p* = 0.041). However, the ABCG2 rs2231142 polymorphism was not associated with sunitinib-induced hypertension or neutropenia (ES = 1.09, 95% CI = 0.69–1.73, *p* = 0.701; ES = 0.87, 95% CI = 0.57–1.31, *p* = 0.501). Compared with the C allele, the ABCB1 rs1128503 T allele was associated with a decreased risk of sunitinib-induced hypertension but worse progression-free survival (PFS) (ES = 0.44, 95% CI = 0.26–0.77, *p* = 0.004; ES = 1.36, 95% CI = 1.07–1.73, *p* = 0.011). There was no significant association between the T allele or C allele of ABCB1 rs1128503 and overall survival (OS) (ES = 0.82, 95% CI = 0.61–1.10, *p* = 0.184). The ABCB1 rs2032582 T allele was associated with worse PFS than the other alleles (ES = 1.46, 95% CI = 1.14–1.87, *p* = 0.003), while there was no significant association between the T allele or other alleles and sunitinib-induced hypertension, HFS, or OS (ES = 0.77, 95% CI = 0.46–1.29, *p* = 0.326; ES = 1.02, 95% CI = 0.65–1.62, *p* = 0.919; ES = 1.32, 95% CI = 0.85–2.05, *p* = 0.215).

**Conclusion:** The results indicate that the ABCG2 rs2231142 polymorphism may serve as a predictor of sunitinib-induced thrombocytopenia and HFS in Asians, while the ABCB1 rs1128503 polymorphism may serve as a predictor of sunitinib-induced hypertension, and both the ABCB1 rs1128503 and rs2032582 polymorphisms may serve as predictors of PFS in RCC. These results suggest a possible application of individualized use of sunitinib according to the genetic background of patients.

## Introduction

Renal cell carcinoma (RCC) is a common cancer with high malignancy, and 20–30% of RCC patients already suffer from metastatic lesions at the time of diagnosis (Basso et al., 2010; Sun et al., 2011). Sunitinib is a first-generation tyrosine kinase inhibitor (TKI) that was approved for the treatment of metastatic RCC (mRCC) in 2006 (Diekstra et al., 2016). In patients with mRCC, sunitinib has been associated with improvements in progression-free survival (PFS) and overall survival (OS) compared with interferon-alpha (Motzer et al., 2009), and it achieves significant response rates in both Western and Japanese patients (Motzer et al., 2006; Tomita et al., 2010). The Metastatic Renal Cell Carcinoma database Consortium International (IMDC) model is the most widely used prognostic models for the prognosis of mRCC in clinical practice and clinical trials (Rini et al., 2018). Currently, sunitinib remains the first-line standard of care for IMDC favorable-risk patients, and for IMDC intermediate- and poor-risk patients, immune checkpoint inhibition, sequencing, and combined systemic therapy have been reported to have an OS benefit (de Velasco et al., 2019; Loo et al., 2019; Stuhler et al., 2020). Increased exposure to sunitinib is associated with improved PFS and OS but also an increased risk for adverse events (Houk et al., 2009; Motzer et al., 2009). Sunitinib-associated toxicities include thrombocytopenia, neutropenia, leucopenia, hand-foot syndrome (HFS), hypertension, mucositis, and diarrhea (Kalantari, 2009; Escudier et al., 2014; Atkins et al., 2015). The therapeutic efficacy and toxicity of sunitinib are very heterogeneous and have been difficult to predict before treatment initiation. Single-nucleotide polymorphisms (SNPs) in genes encoding metabolism enzymes or transporters related to the pharmacokinetics (PK) and pharmacodynamics (PD) of sunitinib have been identified to be associated with the toxicity and efficacy of sunitinib in previous studies (Garcia-Donas et al., 2015; Rodriguez-Antona and Taron, 2015), especially SNPs in genes related to the PK pathways of sunitinib in patients with RCC (Junker et al., 2013; Fiszer and Krzyzosiak, 2014; Narjoz et al., 2015).

Sunitinib is a substrate of ATP-binding cassette member B1 (ABCB1) and another efflux transporter encoded by ATP-binding cassette member G2 (ABCG2) (Low et al., 2016). ABCG2 and ABCB1 are the PK-related genes of sunitinib (Watanabe et al., 2017). The most common functional SNP in ABCG2 was reported to be rs2231142 (421C/A), and those in ABCB1 were rs2032582 (2677G/TA), rs1128503 (1236T/C), and rs1045642 (3435C/T) (Kato et al., 2017; Watanabe et al., 2017; Reustle et al., 2018). ABCG2 rs2231142 was located at Q141K, ABCB1 rs1045642 at | 1154 | , ABCB1 rs1128503 at G412G, and ABCB1 rs2032582 at A893 S/T. The associations of ABCB1 and ABCG2 polymorphisms with sunitinib-induced toxicity and clinical outcomes in patients with RCC have been investigated. However, the associations were controversial. ABCG2 rs2231142 was reported to have no association with thrombocytopenia in Whites and Asians (van Erp et al., 2009; Kato et al., 2017; Zhang et al., 2018). However, in other studies, ABCG2 rs2231142 was associated with severe thrombocytopenia (Low et al., 2016), and patients carrying the ABCG2 rs2231142 AA genotype were more likely to develop thrombocytopenia, neutropenia, and HFS even after adjustment (Kim et al., 2013). Chu et al. reported that the ABCG2 rs2231142 A allele was correlated with a 3-fold decrease in the risk of neutropenia (Chu et al., 2015). Garcia-Donas et al. reported that ABCG2 rs2231142 was not associated with hypertension but seemed to confer protection against HFS, while ABCB1 rs1128503 and rs2032582 seemed to confer protection against hypertension (Garcia-Donas et al., 2011). The ABCB1 rs2032582 TT, AT, and GT genotypes were reported to be significantly correlated with grade 2 and grade 3 HFS (Zhang et al., 2018). However, de Velasco et al. reported that ABCB1 rs2032582 was not associated with sunitinib-induced toxicity (de Velasco et al., 2016). Beuselinck et al. reported that both the PFS and OS of patients with mRCC were significantly associated with SNP rs1128503 in ABCB1 but were not associated with ABCB1 rs2032582 (Beuselinck et al., 2013), while ABCB1 rs1128503 and rs2032582 were not associated with PFS or OS in patients with mRCC in another study (Garcia-Donas et al., 2011).

Identifying the effects of ABCG2 and ABCB1 polymorphisms on sunitinib-induced toxicity and efficacy in patients with RCC could help to optimize the therapeutic management strategy and maximize the clinical benefits of sunitinib. Here we used a meta-analysis to evaluate the associations of ABCG2 rs2231142, ABCB1 rs1128503, and ABCB1 rs2032582 polymorphisms with sunitinib-induced toxicity and those of ABCB1 rs1128503 and ABCB1 rs2032582 polymorphisms with PFS and OS in patients with RCC.

## Methods

### Search Strategy and Selection Criteria

*PubMed, EMBASE, Cochrane Library*, and *Web of Scienc*e were systematically searched using the keywords “sunitinib’’ and ‘‘polymorphisms” to identify eligible studies. Only articles in English were included. The last search was updated on 7 February 2020. We also retrieved eligible studies in the references of all relevant articles. Furthermore, we contact the authors to request missing information to strengthen the analysis.

The inclusion criteria for studies were as follows: 1) studies of RCC patients who received sunitinib treatment; 2) studies evaluating the associations of ABCG2 rs2231142 polymorphism with sunitinib-induced thrombocytopenia, HFS, and hypertension; 3) studies evaluating the associations of ABCB1 rs1128503 and rs2032582 polymorphisms with sunitinib-induced hypertension and HFS; 4) studies evaluating the associations of ABCB1 rs1128503 and rs2032582 polymorphisms with PFS and OS; and 5) studies with available odds ratios (ORs) between genotypes.

The exclusion criteria for studies were as follows: 1) letters, reviews, and case reports; 2) nonclinical studies; and 3) studies that were duplicate publications. In addition, we pooled complete data in the meta-analysis if multiple studies had overlapping data.

### Data Extraction

Two authors independently selected the relevant articles and then extracted the following data from the articles: first author’s name, publication year, study design, sample size, treatment regimen, age, sex, region/ethnicity, ABCG2 rs2231142, ABCB1 rs1128503 and ABCB1 rs2032582 phenotype, genotyping methods, evidence of Hardy-Weinberg equilibrium in cases, adverse events and clinical outcome in ABCG2 rs2231142, ABCB1 rs1128503 and ABCB1 rs2032582 phenotypes, and the ORs of ABCG2 rs2231142 A allele vs. C allele, ABCB1 rs1128503 T allele vs. C allele, and ABCB1 rs2032582 T allele vs. other alleles. If there was any controversy, it was resolved by discussion among the authors.

### Statistical Analysis

STATA software (version 12.0; Stata Corp, College Station, TX, USA) was used to perform the statistical tests. The associations of ABCG2 rs2231142, ABCB1 rs1128503, and ABCB1 rs2032582 polymorphisms with sunitinib-induced toxicity, and ABCB1 rs1128503 and ABCB1 rs2032582 polymorphisms with PFS and OS were evaluated by effect size (ES) with 95% CIs (Cheng et al., 2020). The heterogeneity between studies was assessed by the chi-square-based Q-test and I^2^ tests. Both no-heterogeneity criteria were required to be met (*p* > 0.05 AND I^2^ < 50%) to use the fixed-effects model; otherwise, the random-effects model was used to calculate the pooled ES. The Z test was used to investigate the significance of the ES, and P_Z_ < 0.05 was considered statistically significant. The stability of the results was evaluated by sensitivity analysis. The publication bias among studies was determined by Egger’s and Begg’s tests, and P_E_ < 0.05 was considered significant.

## Results

### Study Selection

**FIGURE 1:**
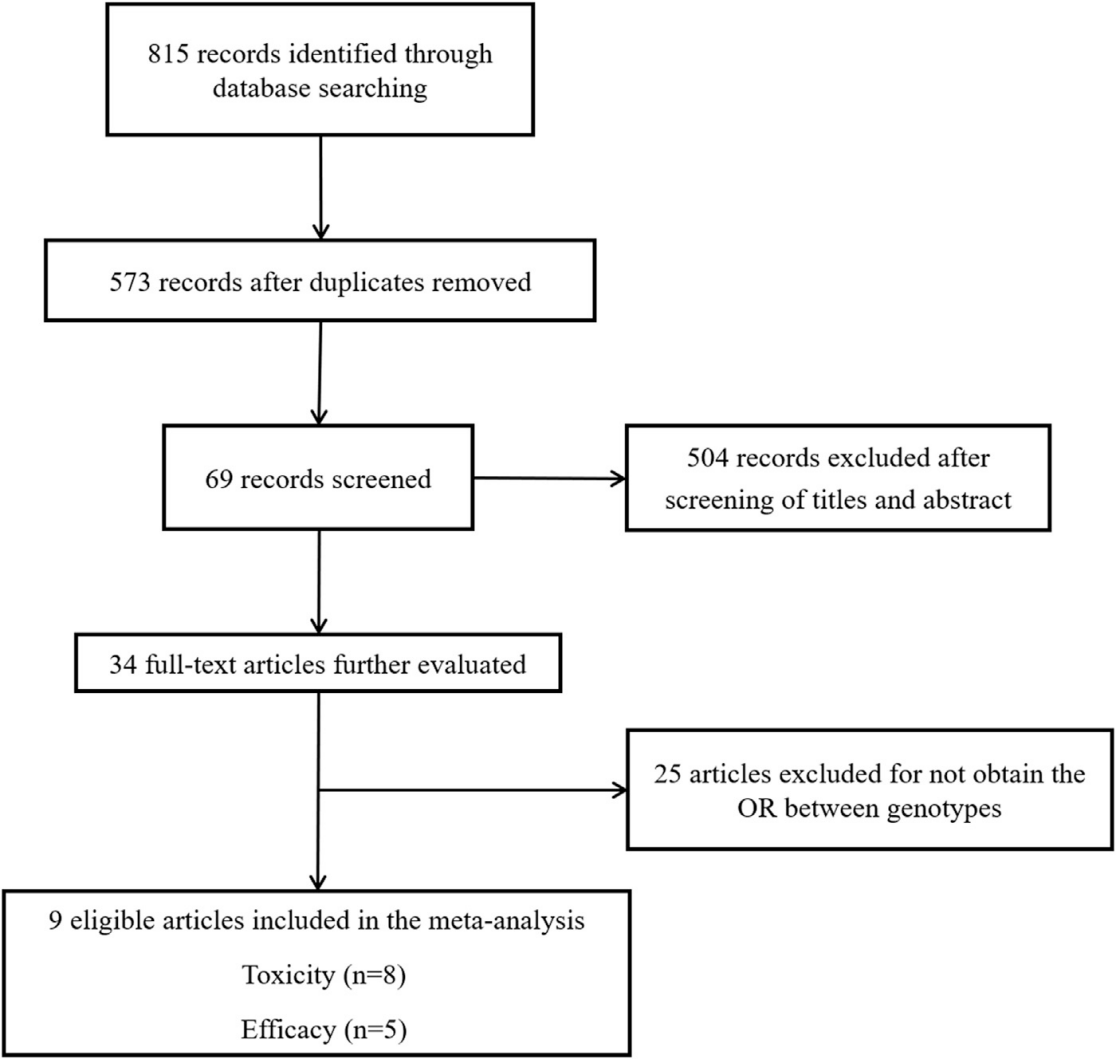
Flow diagram of the study selection process.

The study selection process is shown in Figure 1. We initially retrieved 815 articles from electronic databases, including *PubMed, EMBASE, Science Direct*, and *Web of Science*. We included 34 relevant studies after removing the duplicates and reviewing the titles, abstracts, and full texts. Then, 25 articles were excluded because they did not determine the ORs between genotypes. Ultimately, 8 articles published between 2011 and 2018 assessing the relationships of ABCG2 rs2231142, ABCB1 rs1128503, and ABCB1 rs2032582 polymorphisms with sunitinib-induced toxicity, and 5 articles published between 2011 and 2017 assessing the relationships of ABCB1 rs1128503 and ABCB1 rs2032582 polymorphisms with PFS and OS were included in the current meta-analysis (Garcia-Donas et al., 2011; Beuselinck et al., 2013; Kim et al., 2013; Diekstra et al., 2015; Chu et al., 2015; de Velasco et al., 2016; Low et al., 2016; Numakura et al., 2017; Zhang et al., 2018).

### Characteristics of the Included Studies

The main characteristics of all eligible studies are shown in Tables 1–3. In total, 9 studies including 1081 patients were enrolled in the pooled analysis. The total sample size is the sum of the sample in each study except for Garcia-Donas et al. study, because patients in the study by Diekstra et al. were pooled from 5 studies including study of Garcia-Donas et al., and we only used data from Garcia-Donas et al. when those from Diekstra et al. did not indicate the specific toxicity or efficacy. The included studies are all observational. Five studies were prospective cohort studies, and four studies were retrospective cohort studies. Five studies (55.6%) reported on Asian individuals, and 4 (44.4%) studies reported mainly on Caucasian individuals. Sunitinib was given as monotherapy in the included studies. The variant allele A frequency of ABCG2 rs2231142 in Asians was significantly higher than that in Caucasians (34.1% vs. 5.3%), the variant allele C frequency of ABCB1 rs1128503 in the two different ethnic groups was similar (37.6% vs. 31.1%), and the variant AT genotype frequency of ABCB1 rs2032582 in Asians was a little higher than that in Caucasians (29.9% vs. 16.5%) in the included studies. The selected results contained the necessary information according to the STrengthening the REporting of Genetic Association Studies (STREGA) (Little et al., 2009).

**TABLE 1:** Characteristics of studies included in the meta-analysis.

Study	Year	Study design	Sample size	Treatment regimen	Age (year)	Male (%)	Region/ethnicity	ABCG2 rs2231142 phenotype (CC/CA/AA)	ABCB1 rs1128503 (TT/CT/CC)	ABCB1 rs2032582 (GG/GT ± GA/TT+AT)	Genotyping method	Hardy-Weinberg equilibrium
Diekstra et al. (2015)	2015	Retrospective	330	Sunitinib 50, 37.5, or 25 mg/day	61 (55,69)	69	White 321, black 5, Asian 3, Latin American 1, Arab 3	-	-	-	KASPar SNP	Yes
Chu et al. (2015)	2015	Retrospective	97	Sunitinib 50 or 37.5 mg/day	58 (18–79)	77.3	Chinese 86, Malay 7, Indian 4	50/38/7	35/49/9	25/44/27	PCR	Yes
Garcia-Donas et al. (2011)	2011	Prospective	95	Sunitinib 50, 37.5, or 25 mg/day	65 (42–87, 56–73)	68	Caucasian	85/10/0	36/45/14	38/39/15	KASPar SNP genotyping system	Yes
Kim et al. (2013)	2013	Retrospective	65	Sunitinib 50 mg/day	59 (36–81)	78.5	Korean	-	-	-	TaqMan SNP genotyping assays	Yes
Low et al. (2016)	2016	Prospective	219	Sunitinib	63 (82–83)	73.5	Japanese	-	-	-	PCR	-
Numakura et al. (2017)	2017	Prospective	70	Sunitinib 50 or 37.5 mg/day	64 (28–85)	79	Japanese	CC 33, CA ± AA 31	CT ± TT 52, CC 12	GG 27, others 37	PCR-restriction fragment length polymorphism method	Yes
de velasco et al. (2016)	2016	Prospective	159	Sunitinib	61 (53–67)	72	Caucasian	-	-	50/71/32	iPlex gold platform	Yes
Zhang et al. (2018)	2018	Prospective	53	Sunitinib 50, 37.5, or 25 mg/day	52 (45–61)	75.47	Chinese	19/24/10	27/23/3	10/26/17	LifePro thermal cycler	Yes
Beuselinck et al. (2013)	2013	Retrospective	88	Sunitinib	59 (38–84)	68	Caucasian 83/unknown 5	-	38/35/15	32/36/12	High-throughput SNP genotyping	-

“-“no description.

**TABLE 2:** Data for studies included in the meta-analysis of sunitinib-induced toxicities.

Study	Year	Thrombocytopenia (OR, 95% CI)	Hypertension (OR, 95% CI)			Hand-foot syndrome (OR, 95% CI)		Neutropenia (OR, 95% CI)
		ABCG2 rs2231142 (A allele vs. C allele)	ABCG2 rs2231142 (A allele vs. C allele)	ABCB1 rs1128503 (T allele vs. C allele)	ABCB1 rs2032582 (T allele vs. other alleles)	ABCG2 rs2231142 (A allele vs. C allele)	ABCB1 rs2032582 (T allele vs. other alleles)	ABCG2 rs2231142 (A allele vs. C allele)
Diekstra et al. (2015)	2015	-	0.03 (0.001, 0.85)	-	-	-	-	-
Chu et al. (2015)	2015	-	-	-	-	-	-	0.3 (0.1, 0.9)
Garcia-Donas et al. (2011)	2011	-	-	0.41 (0.20, 0.81)	0.42 (0.21, 0.84)	0.11 (0.01, 0.92)	1.00 (0.55, 1.82)	-
Kim et al. (2013)	2013	1.74 (0.81, 3.75)	-	-	-	6.76 (2.16, 21.13)	1.11 (0.37, 3.28)	1.89 (0.76, 4.75)
Low et al. (2016)	2016	1.856 (1.172, 2.939)	1.23 (0.713, 2.124)	-	-	1.24 (0.776, 1.98)	-	0.856 (0.512, 1.431)
Numakura et al. (2017)	2017	0.73 (0.26, 2.05)	0.66 (0.24, 1.80)	0.63 (0.18, 2.21)	5.37 (1.06, 9.52)	1.08 (0.36, 4.73)	3.17 (1.02, 14.61)	-
de velasco et al. (2016)	2016	-	-	-	0.57 (0.13, 2.46)	-	-	-
Zhang et al. (2018)	2018	2.3 (0.4, 12.0)	4.4 (0.7, 26.5)	0.4 (0.1, 1.4)	0.5 (0.1, 2.0)	1.4 (0.2, 7.9)	0.3 (0.1, 1.6)	-

“-“no description.

**TABLE 3:** Data for studies included in the meta-analysis of sunitinib efficacy.

Study	Year	Progression-free survival (HR, 95% CI)		Overall survival (HR, 95% CI)	
		ABCB1 rs1128503 (T allele vs. C allele)	ABCB1 rs2032582 (T allele vs. other alleles)	ABCB1 rs1128503 (T allele vs. C allele)	ABCB1 rs2032582 (T allele vs. other alleles)
Diekstra et al. (2015)	2015	1.42 (1.07, 1.90)	1.36 (1.03, 1.81)	0.66 (0.46, 0.94)	-
Garcia-Donas et al. (2011)	2011	-	-	-	1.47 (0.85, 2.54)
Numakura et al. (2017)	2017	5.8 (0.87, 7.51)	2.03 (0.74, 2.57)	0.36 (0.31, 7.63)	0.33 (0.29, 3.74)
Chu et al. (2015)	2015	1.7 (0.9, 3.2)	1.5 (0.5, 4.7)	1.7 (1.0, 3.1)	2.0 (0.8, 5.0)
Beuselinck et al. (2013)	2013	0.464 (0.234, 0.918)	**-**	0.415 (0.193, 0.894)	**-**

“-“no description.

### Relationship Between the ABCG2 rs2231142 Polymorphism and Toxicity

**FIGURE 2:**
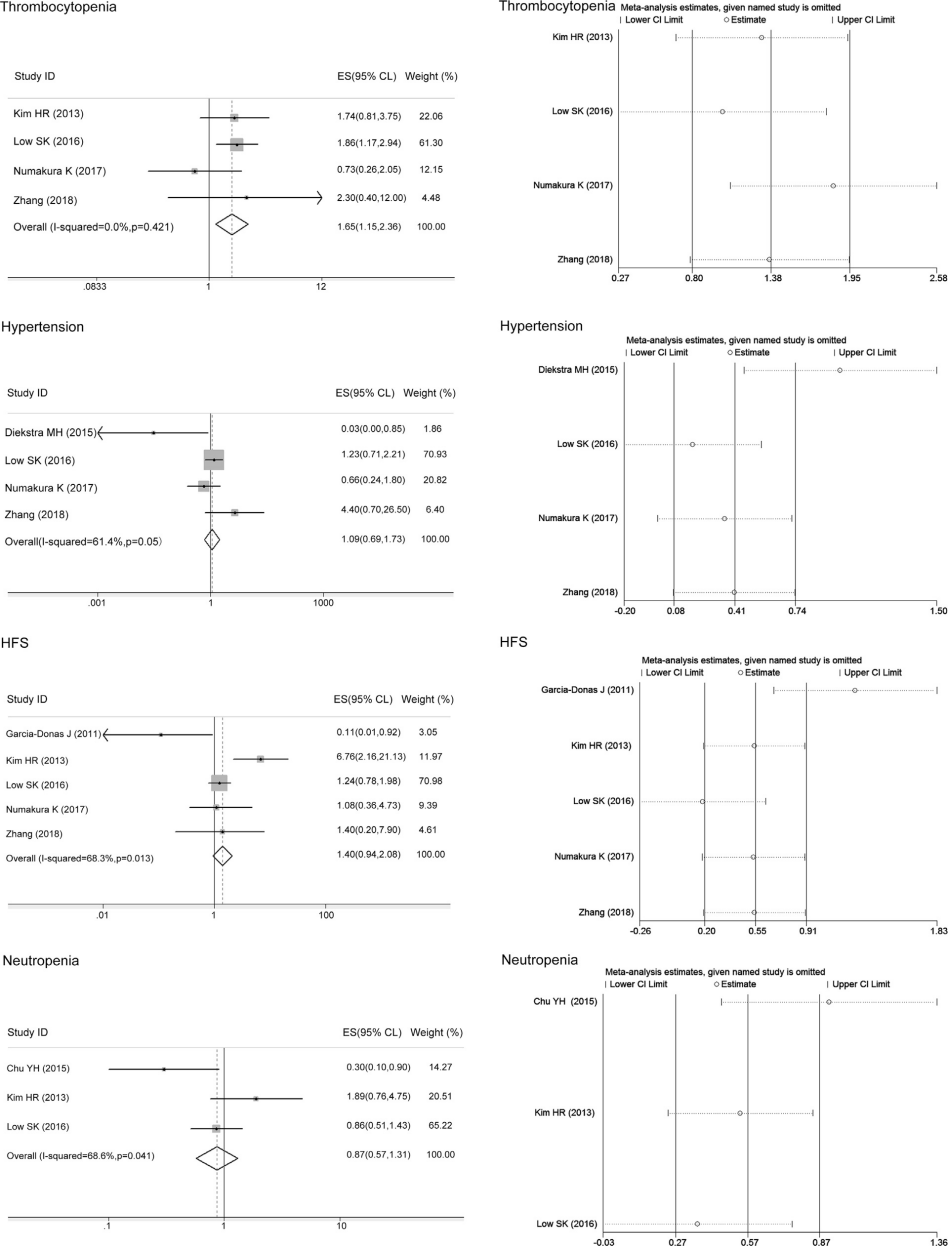
Forest plots estimating the associations of ABCG2 rs2231142 polymorphism with sunitinib-induced toxicity and the corresponding sensitivity analysis (A allele vs. C allele). The ABCG2 rs2231142 A allele was significantly associated with an increased risk of thrombocytopenia in Asians compared with the C allele, while there were no significant associations between the A allele and C allele in hypertension, HFS, or neutropenia. The sensitivity analysis showed that no single study qualitatively altered the pooled ES of thrombocytopenia and neutropenia; however, the study by Diekstra et al. altered the hypertension results, and the study by Garcia-Donas et al. altered the HFS results.

**FIGURE 3:**
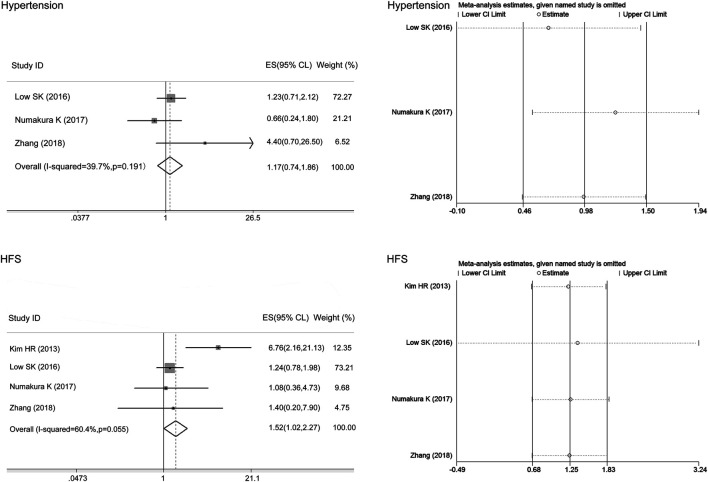
Forest plots estimating the associations of the ABCG2 rs2231142 polymorphism with sunitinib-induced toxicities in Asians and the corresponding sensitivity analysis (A allele vs. C allele). Compared with the C allele, the ABCG2 rs2231142 A allele was significantly associated with an increased risk of HFS in Asians. There were no significant associations between the A allele and C allele in hypertension in this population. The sensitivity analysis showed that no single study qualitatively altered the pooled ES.

Thinking the consistency of the overall results, we initially analyzed the associations of the ABCG2 polymorphisms with sunitinib-induced toxicities without regard to ethnic background. The meta-analysis results are shown in Table 4. There was no heterogeneity between studies assessing the relationships of the ABCG2 rs2231142 polymorphism with sunitinib-induced thrombocytopenia (I^2^ = 0, *p* > 0.05), and the fixed-effects model was applied to the analysis. There was heterogeneity between studies assessing the relationships of ABCG2 rs2231142 polymorphism with sunitinib-induced hypertension, HFS, and neutropenia (I^2^ = 61.4%, 68.3%, and 68.6%, respectively), and the random-effects model was applied to the analysis. Based on the results, we found that compared with the C allele, the ABCG2 rs2231142 A allele was significantly associated with an increased risk of sunitinib-induced thrombocytopenia in Asians (ES = 1.65, 95% CI = 1.15–2.36, *p* = 0.006; Figure 2), while there were no significant associations between the A allele and C allele in sunitinib-induced hypertension, HFS, or neutropenia (ES = 1.09, 95% CI = 0.69–1.73, *p* = 0.701; ES = 1.40, 95% CI = 0.94–2.08, *p* = 0.094; ES = 0.87, 95% CI = 0.57–1.31, *p* = 0.501; Figure 2). Since the patients included in the meta-analysis of thrombocytopenia and neutropenia were all Asian (Table 2), we only conducted a subgroup analysis of HFS and hypertension in Asians. Compared with the C allele, the ABCG2 rs2231142 A allele was significantly associated with an increased risk of sunitinib-induced HFS (ES = 1.52, 95% CI = 1.02–2.27, *p* = 0.041; Figure 3). There was also no significant associations between the A allele and C allele in sunitinib-induced hypertension in Asians (ES = 1.17, 95% CI = 0.74–1.86, *p* = 0.504; Figure 3).

**TABLE 4:** Summary of the meta-analysis and publication bias.

Allele	No. of studies	Heterogeneity P value	I^2^ value (%) for heterogeneity test	Model	ES (95%CI)	P Value	Z	P Value for Egger’s (Begg’s) bias test
ABCG2 rs2231142 (A allele vs. C allele)								
Hand-foot syndrome	5	0.013	68.3	R	1.40 (0.94, 2.08)	0.094	1.67	0.737 (1.000)
Hand-foot syndrome (Asian)	4	0.055	60.4	R	1.52 (1.02, 2.27)	0.041	2.04	-
Thrombocytopenia (Asian)	4	0.421	0	F	1.65 (1.15, 2.36)	0.006	2.72	0674 (1.000)
Hypertension	4	0.051	61.4	R	1.09 (0.69, 1.73)	0.701	0.38	-
Hypertension (Asian)	3	0.191	39.7	F	1.17 (0.74, 1.86)	0.504	0.67	-
Neutropenia (Asian)	3	0.041	68.6	R	0.87 (0.57, 1.31)	0.501	0.67	-
ABCB1 rs1128503 (T allele vs. C allele)								
Hypertension	3	0.830	0	F	0.44 (0.26, 0.77)	0.004	2.87	-
PFS	4	0.001	82.4	R	1.36 (1.07, 1,73)	0.011	2.54	0.155 (0.296)
OS	4	0.024	68.3	R	0.82 (0.61, 1.10)	0.184	1.33	-
ABCB1 rs2032582 (T allele vs. other alleles)								
Hypertension	4	0.001	80.6	R	0.77 (0.46, 1.29)	0.326	0.98	-
Hand-foot syndrome	4	0.121	48.4	F	1.02 (0.65, 1.62)	0.919	0.10	-
PFS	3	0.516	0	F	1.46 (1.14, 1.87)	0.003	2.95	0.536 (1.000)
OS	3	0.065	63.3	R	1.32 (0.85, 2.05)	0.215	1.24	-

“-” not available.

### Correlations of the ABCB1 rs1128503 Polymorphism With Toxicity and Clinical Outcomes

**FIGURE 4:**
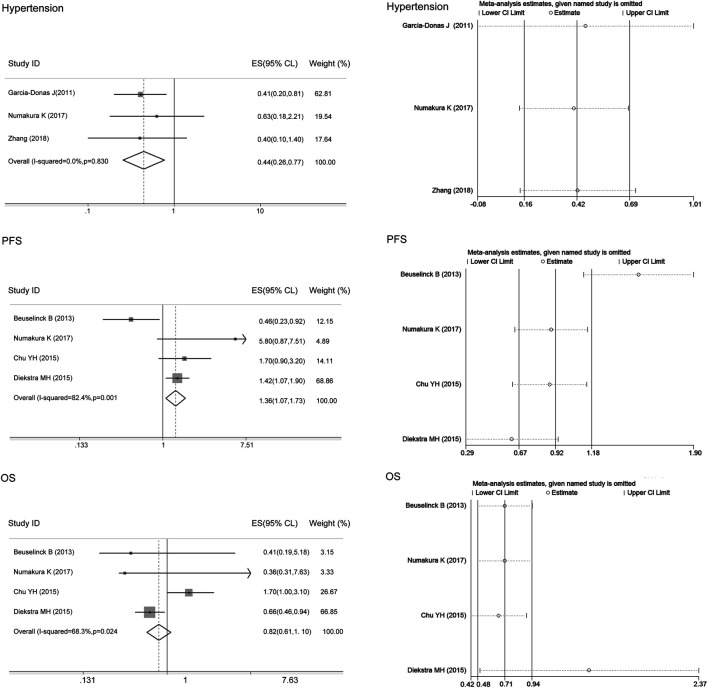
Forest plots estimating the associations of the ABCB1 rs1128503 polymorphism with sunitinib-induced hypertension, progression-free survival (PFS), and overall survival (OS), and the corresponding sensitivity analysis (T allele vs. C allele). Compared with the C allele, the ABCB1 rs1128503 T allele was significantly associated with a decreased risk of hypertension and worse PFS. There was no significant association between the T allele and C allele in OS. The sensitivity analysis showed that no single study qualitatively altered the pooled ES of hypertension; however, the study by Beuselinck et al. altered the PFS results, and the study by Diekstra et al. altered the OS results.

We only performed the meta-analysis of rs1128503 in ABCB1 in mixed ethnicity as the variant allele C frequency in Asians and Caucasians was similar. There was no heterogeneity between studies assessing the associations of the ABCB1 rs1128503 polymorphism with sunitinib-induced hypertension (I^2^ = 0, *p* > 0.05), and the fixed-effects model was applied to the analysis. There was heterogeneity between studies assessing the relationship of ABCB1 rs1128503 polymorphism with sunitinib-induced PFS and OS (I^2^ = 82.4%, 68.3%, *p* < 0.05), and the random-effects model was applied to the analysis. The results showed that compared with the C allele, the ABCB1 rs1128503 T allele was significantly associated with a decreased risk of sunitinib-induced hypertension and worse PFS (ES = 0.44, 95% CI = 0.26–0.77, *p* = 0.004; ES = 1.36, 95% CI = 1.07–1.73, *p* = 0.011; Figure 4). There was no significant association between the T allele and C allele in sunitinib-induced OS (ES = 0.82, 95% CI = 0.61–1.10, *p* = 0.184; Figure 4).

### Correlations of the ABCB1 rs2032582 Polymorphism With Toxicity and Clinical Outcomes

**FIGURE 5:**
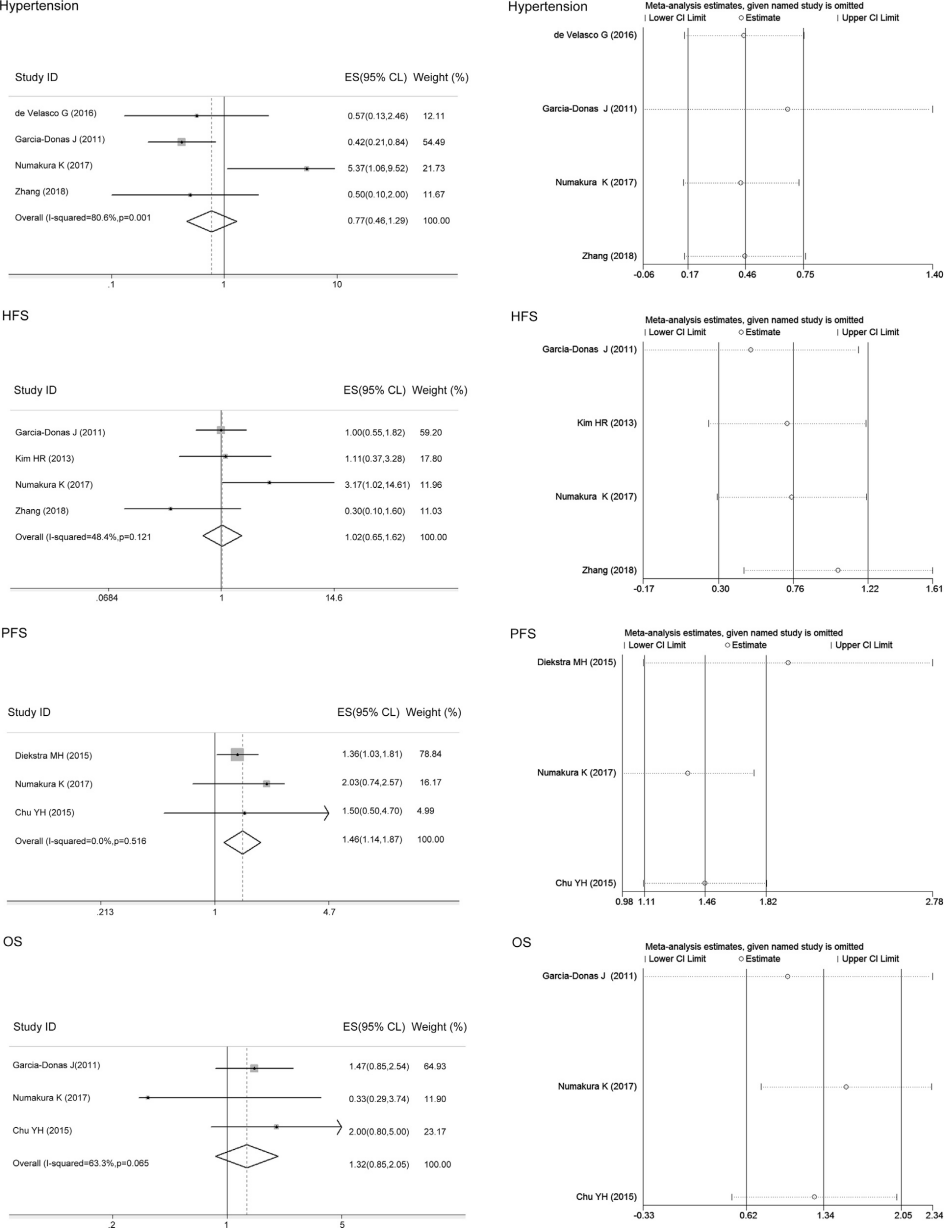
Forest plots estimating the associations of the ABCB1 rs2032582 polymorphism with sunitinib-induced hypertension, hand-foot syndrome (HFS), progression-free survival (PFS), and overall survival (OS), and the corresponding sensitivity analysis (T allele vs. other alleles). Compared with the other alleles, the ABCB1 rs2032582 T allele was significantly associated with worse PFS. In contrast, there was no significant association between the T allele and other alleles in sunitinib-induced hypertension, HFS, or OS. The sensitivity analysis showed that no single study qualitatively altered the pooled ES.

There was heterogeneity between studies assessing the associations of the ABCB1 rs2032582 polymorphism with sunitinib-induced hypertension and OS (I^2^ = 80.6%, 63.3%), and the random-effects model was applied to the analysis. There was no heterogeneity between studies assessing the relationship of the ABCB1 rs2032582 polymorphism with sunitinib-induced HFS or PFS (I^2^ = 48.4%, 0, *p* > 0.05), and the fixed-effects model was applied to the analysis. The results showed that compared with the other alleles, the ABCB1 rs2032582 T allele was significantly associated with worse PFS (ES = 1.46, 95% CI = 1.14–1.87, *p* = 0.003; Figure 5). In contrast, there was no significant association between the T allele and other alleles in sunitinib-induced hypertension, HFS, or OS (ES = 0.77, 95% CI = 0.46–1.29, *p* = 0.326; ES = 1.02, 95% CI = 0.65–1.62, *p* = 0.919; ES = 1.32, 95% CI = 0.85–2.05, *p* = 0.215; Figure 5).

### Sensitivity Analysis and Publication Bias

The sensitivity analysis in the meta-analysis of the relationships of the ABCG2 rs2231142 polymorphism with sunitinib-induced thrombocytopenia and neutropenia showed that no single study qualitatively altered the pooled ES, however, the study by Diekstra et al. may qualitatively altered the result of hypertension and that by Garcia-Donas et al. may qualitatively altered the result of HFS (Figure 2). When we omitted the article, the meta-analysis results for HFS changed but not the hypertension results, and no single study qualitatively altered the pooled ES in either analysis, which provided evidence of the stability of the meta-analysis (Figure 3). There was no publication bias for thrombocytopenia and HFS (*p* > 0.05, Table 4).

**FIGURE 6:**
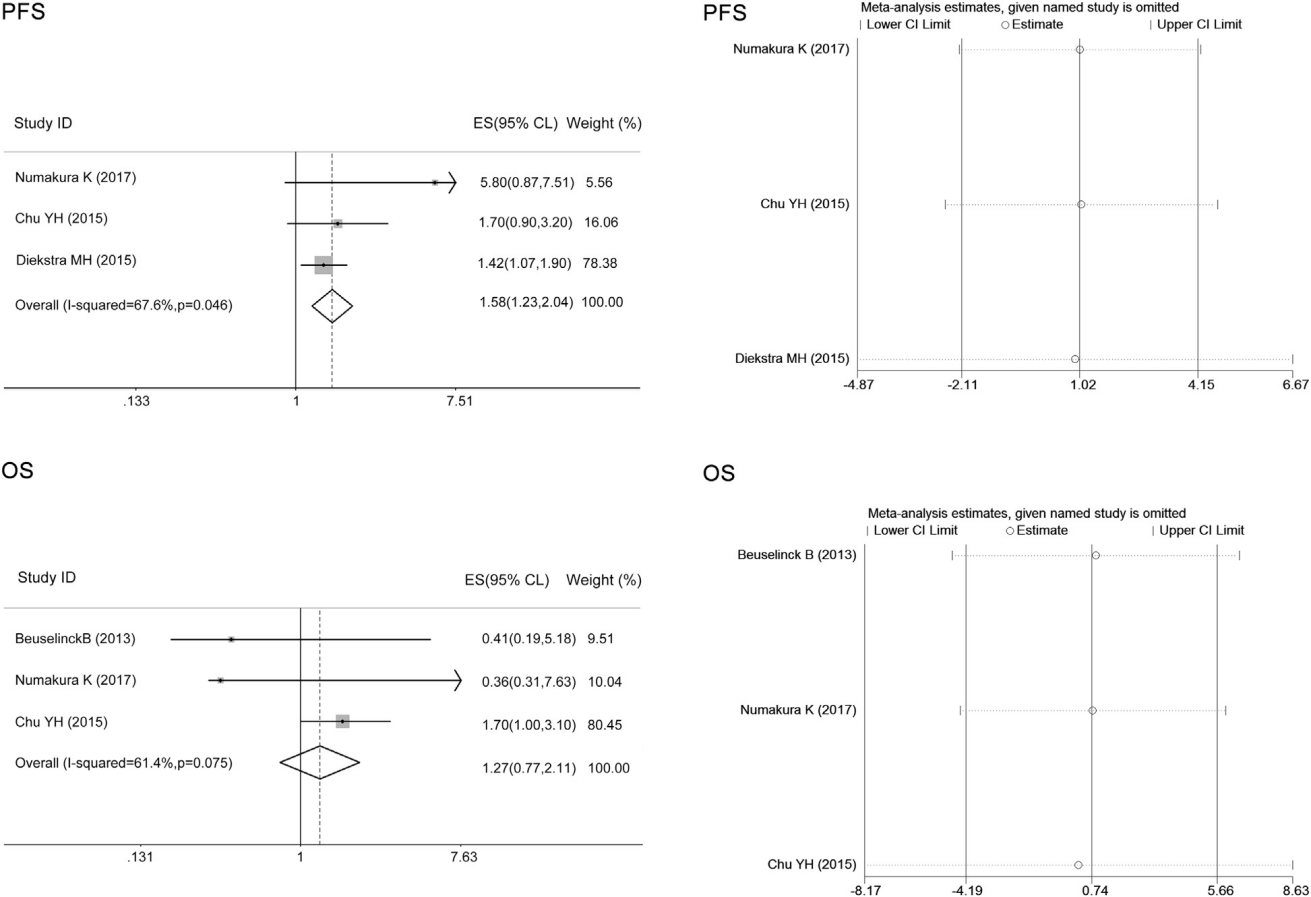
Forest plots estimating the associations of ABCB1 rs1128503 polymorphism with sunitinib-induced progression-free survival (PFS) and overall survival (OS) when we omitted the article that altered the pooled ES (T allele vs. C allele). The ABCB1 rs1128503 polymorphism was associated with PFS but not OS, and no single study qualitatively altered the pooled ES in both analysis.

The sensitivity analysis in the meta-analysis of the relationships of the ABCB1 rs1128503 polymorphism with sunitinib-induced hypertension showed that no single study qualitatively altered the pooled ES; however, the study by Beuselinck et al. may qualitatively altered the result of PFS and that by Diekstra et al. may qualitatively altered the result of OS (Figure 4). When we omitted the article, the meta-analysis results for PFS (ES = 1.58, 95% CI = 1.23–2.04, *p* < 0.001) and OS (ES = 1.27, 95% CI = 0.77–2.11, *p* = 0.353) were not changed, and no single study qualitatively altered the pooled ES in either analysis (Figure 6).

The sensitivity analysis in the meta-analysis of the relationships of the ABCB1 rs2032582 polymorphism with sunitinib-induced hypertension, HFS, PFS, and OS showed that no single study qualitatively altered the pooled ES (Figure 5). There was no publication bias for PFS in ABCB1 rs1128503, or ABCB1 rs2032582 polymorphisms (*p* > 0.05, Table 4).

## Discussion

In the current meta-analysis, we initially analyzed the associations of the ABCG2 rs2231142 polymorphism with sunitinib-induced thrombocytopenia, HFS, hypertension, and neutropenia; the associations of the ABCB1 rs1128503 polymorphism with sunitinib-induced hypertension, PFS, and OS; and the associations of ABCB1 rs2032582 polymorphisms with sunitinib-induced hypertension, HFS, PFS, and OS, without regard to ethnic background. However, we found heterogeneity between studies based on the results. We further considered the origin of the heterogeneity, and we found that the heterogeneity might derive from ethnicity as the variant allele A frequency of ABCG2 rs2231142 in Asians was significantly higher than that in Caucasians, then we conducted a subgroup analysis of hypertension and HFS in Asians. Next, we did the sensitivity analysis of all meta-analysis to evaluate their stability. The results showed that one article with population Caucasians altered the results of HFS in ABCG2, which indicated that the heterogeneity might derive from ethnicity. For ABCB1 rs1128503 and ABCB1 rs2032582 results, there were no article qualitatively altered the results. The sensitivity analysis results provided evidence of the stability of the meta-analysis.

The therapeutic efficacy of sunitinib is heterogeneous among different IMDC risk groups (Rini et al., 2018). In studies included in the meta-analysis, they did not mention the IMDC stratification, and we could not analyze the effect of IMDC on toxicity and clinical outcomes. The treatment regimen of sunitinib in the included articles was not completely consistent, and five studies had three starting dose (50 or 37.5 or ≤37.5, mg/day) (Garcia-Donas et al., 2011; Diekstra et al., 2015; Chu et al., 2015; Numakura et al., 2017; Zhang et al., 2018). In Chu et al. study, the starting dose (50 or 37.5 or ≤37.5, mg/day) was not associated with toxicities, including leukopenia, neutropenia, or diarrhea but was associated with PFS and OS (Chu et al., 2015), and we used the results of multivariate analysis in the meta-analysis, which showed no significant association. Zhang et al. study showed no significant association between toxicity of grade <3 or ≥3 and each dosage (25, 37.5, and 50 mg/d) (Zhang et al., 2018). The effect of dose on toxicities and clinical outcome were not mentioned in the other three studies (Garcia-Donas et al., 2011; Diekstra et al., 2015; Numakura et al., 2017). RCC is more common in men. However, the included studies did not investigate the differences between these genetic polymorphisms in different genders. In the included studies, Chu et al. reported that the gender was not associated with the leucopenia, neutropenia, diarrhea, PFS or OS (Chu et al., 2015). In Diekstra et al. study, gender seemed to show association with thrombocytopenia and toxicity grade >2 in univariate analysis, however, there was no association in the multivariate analysis (Diekstra et al., 2015). Zhang et al. showed that gender was associated with hypertension in univariate analysis, but the results of multivariate analysis showed no significant association (Zhang et al., 2018). In Low et al. study, gender was only associated with leucopenia (Low et al., 2016), which was not included in the meta-analysis. In summary, we used ORs of multivariate analysis in the pooled ES analysis, excluding other possible factors affecting toxicity and efficacy of sunitinib, such as gender, age, sunitinib dosage, laboratory indexes, and other related gene polymorphisms in each included study, which strengthened our conclusions.

Compared with non-Asians, Asians are more likely to develop sunitinib-induced adverse effects (Kim et al., 2011; Lee et al., 2014). The incidence of sunitinib-induced hematotoxicity in Japanese patients with RCC is higher when compared to European populations (Low et al., 2016; Kato et al., 2017). The AA genotype of rs2231142 in ABCG2 has been associated with an increase in systemic exposure to sunitinib, possibly causing sunitinib-related adverse events. In the current meta-analysis, we found that compared with the C allele, the ABCG2 rs2231142 A allele was significantly associated with an increased risk of sunitinib-induced thrombocytopenia in Asians. However, we could not find associations of ABCG2 rs2231142 polymorphisms with hypertension and neutropenia in the meta-analysis.

Garcia-Donas et al. reported that the A allele of ABCG2 rs2231142 seemed to confer protection against HFS in Caucasians (Garcia-Donas et al., 2011), while in the other four studies carried out in Asians, an increased risk of development of HFS was found (Kim et al., 2013; Low et al., 2016; Numakura et al., 2017; Zhang et al., 2018). In our results, there was no significant association between the A allele and C allele in sunitinib-induced HFS. However, when we conducted a subgroup analysis in Asians, we found that compared with the C allele, the ABCG2 rs2231142 A allele was significantly associated with an increased risk of sunitinib-induced HFS. A possible reason for this difference might be the small number of patients with homozygous alleles among Caucasians. The ABCG2 rs2231142 A allele is located within the ATP-binding cassette domain that regulates the ATP binding activity of ABCG2 protein, which reduces ATPase activity (Mizuarai et al., 2004), induces the reduction of transport activity, increases drug accumulation, decreases the efflux velocity of the drug (Imai et al., 2002), and thus increases the systemic exposure to sunitinib and is subsequently more likely to lead to the development of toxicity (Mizuno et al., 2012). It has been reported that the variant allele A of ABCG2 rs2231142 in Asians is approximately 30%, in Caucasians is approximately 10%, and in African Americans is approximately 5% (Diekstra et al., 2016). Diekstra et al. reported that the ABCG2 rs2231142 polymorphism was significantly associated with sunitinib-induced dose reduction after cycle 1, 2, or 3 (Diekstra et al., 2015). Although other previous studies have reported that the ABCG2 rs2231142 polymorphism is not significantly associated with toxicity-related dose reduction (Garcia-Donas et al., 2011; Kato et al., 2017; Zhang et al., 2018) or time to dose reduction in mRCC patients (Numakura et al., 2017), further studies should verify whether dose adjustment based on early onset thrombocytopenia and HFS in Asians prolongs sunitinib treatment.

Patients carrying the variant genotypes of rs1128503 and rs2032582 in ABCB1 showed an increased clearance of sunitinib and its active metabolite SU12662, consequently leading to lower exposure to sunitinib, reduced hypertension, and decreases in PFS and OS (Garcia-Donas et al., 2011; Beuselinck et al., 2014; Diekstra et al., 2014; Teo et al., 2015). In the current meta-analysis, we found that compared with the C allele, the ABCB1 rs1128503 T allele was significantly associated with a decreased risk of sunitinib-induced hypertension but worse PFS in Asians and Caucasians; and the ABCB1 rs2032582 T allele was significantly associated with worse PFS compared with other alleles, which was consistent with previous studies (Diekstra et al., 2015; Chu et al., 2015; Numakura et al., 2017). However, there was no significant association between the ABCB1 rs2032582 T and G alleles in sunitinib-induced hypertension, HFS, or OS. Four included studies also investigated the association of haplotype ABCB1 (rs1128503, rs2032582, rs1045642) with sunitinib induced toxicities and clinical outcome. In Kim et al. study, there were no significant differences among CGC, TTT, and TGC haplotype of ABCB1 (rs1128503, rs2032582, rs1045642) in thromobocytopenia, neutropenia, anemia, or HFS (Kim et al., 2013). Diekstra et al. reported that PFS and OS were improved in the presence of CGT in haplotype ABCB1 (rs1128503, rs2032582, rs1045642) (Diekstra et al., 2015), however, there was no significant difference in median PFS between the present haplotype and absent haplotype in another study (Beuselinck et al., 2013). In Chu et al. study, ABCB1 rs1045642, 1236 rs1128503, rs2032582 TTT haplotype was correlated with a 10-fold (*p* = 0.03) decrease in the risk of neutropenia and inferior PFS and OS (Chu et al., 2015). We should also pay attention to patients carrying the opposite haplotype.

SNPs in sunitinib target candidate genes, including vascular endothelial growth factor receptors (VEGFRs) 1, 2, and 3; Fms-like tyrosine kinase 3 receptor (FLT3); and PK-related genes, including cytochrome P450 1A1 (CYP1A1), CYP3A5, NR1/2, and NR1/3, have also been reported to be associated with sunitinib-induced toxicity and efficacy in patients with mRCC (van Erp et al., 2009; Garcia-Donas et al., 2011; van der Veldt et al., 2011; Eechoute et al., 2012; Kim et al., 2012; Beuselinck et al., 2013; Beuselinck et al., 2014; Kato et al., 2017; Watanabe et al., 2017). SNPs rs2010963 and rs2070744 in VEGF were reported to be associated with increased chances for the occurrence and duration of hypertension (Eechoute et al., 2012; Kim et al., 2012). The FLT3 738 T/C polymorphism and the CYP3A5 A allele (CYP3A5*1) of rs776746 were reported to be associated with dose reduction due to sunitinib-induced toxicity (Garcia-Donas et al., 2011; Kato et al., 2017). Van Erp et al. observed an increased risk for leukopenia in CYP1A1 rs1048943 and FLT3 rs1933437 and an absence of CAG in the NR1/3 haplotype (rs2307424, rs2307418, rs4073054); CYP1A1 rs1048943 was associated with mucosal inflammation; and EGFR2 rs2305948 was associated with any toxicity > grade 2 (van Erp et al., 2009). Watanabe A et al. reported that the STAT3 polymorphism contributes to a risk factor for stomatitis (Watanabe et al., 2017). The rs9582036, rs9582036, and rs9554320 in VEGF-R1; rs2981582 and rs2305948 in VEGF-R2; rs307826 and rs307821 in VEGF-R3; and rs3025039 in VEGF-A were reported to be associated with PFS and OS in mRCC patients (Garcia-Donas et al., 2011; Kim et al., 2012; Beuselinck et al., 2013; Beuselinck et al., 2014). Compared with the disadvantageous genetic profile carriers, carriers of the genetic profile (at least an A allele in CYP3A5, a TCG copy in ABCB1, or a missing CAT copy in NR1/3) showed improved PFS and OS (van der Veldt et al., 2011). SNP rs2276707 in NR1/2 and SNPs rs2307424 and rs4073054 in NR1/3 were also reported to be associated with PFS and OS in patients with mRCC (Beuselinck et al., 2013).

We did not include rs1045642 in ABCB1 in the meta-analysis because we could not obtain the ORs between the genotypes or because the included article numbers were below three. ABCG2 polymorphisms have also been reported to be associated with other sunitinib-induced toxicity in mRCC patients, such as hypothyroidism (Werbrouck et al., 2018). The T alleles of ABCB1 rs1128503 and ABCB1 rs2032582 were reported to be correlated with decreases in the risk of neutropenia and diarrhea, respectively (Chu et al., 2015). We did not include all sunitinib-induced toxicities or SNPs associated with sunitinib-induced toxicity and efficacy for the above reason, or there was no controversy.

To our knowledge, the present study is the first meta-analysis to investigate the associations of ABCB1 and ABCG2 polymorphisms with sunitinib-induced toxicity and efficacy in patients with RCC. Based on our results, compared with the C allele, the ABCG2 rs2231142 allele A was significantly associated with increased risks of sunitinib-induced thrombocytopenia and HFS in Asians, while the T alleles of ABCB1 rs1128503 and ABCB1 rs2032582 were significantly associated with worse PFS than the other alleles. The results indicate that the ABCG2 rs2231142 polymorphism may serve as a predictor of sunitinib-induced thrombocytopenia and HFS in Asians, while the ABCB1 rs1128503 polymorphism may serve as a predictor of sunitinib-induced hypertension, and both the ABCB1 rs1128503 and rs2032582 polymorphisms may serve as predictors of PFS in RCC. Although our analysis showed no significant relationships of ABCB1 and ABCG2 polymorphisms with other sunitinib-induced severe toxicity or OS, we should also pay more attention to the use of sunitinib in Asian patients. The results may support the possible application of individualized use of sunitinib according to the genetic background of patients with RCC. Genotyping for ABCG2 rs2231142, ABCB1 rs1128503 and rs2032582 polymorphism could become a clinical routine practice to select the appropriate dose to decrease the risk of sunitinib-induced thrombocytopenia and HFS in Asians while ensuring efficacy. The population pharmacokinetic model based on the genotypes should be established to predict the occurrence of sunitinib-induced toxicities and the efficacy.

## Data Availability

The original contributions presented in the study are included in the article/Supplementary Material, further inquiries can be directed to the corresponding author.
